# Metastatic Prostatic Adenocarcinoma Masquerading as Pulmonary Tuberculosis: A Case Report

**DOI:** 10.7759/cureus.24104

**Published:** 2022-04-13

**Authors:** Mona Vohra, Ajay Lanjewar, Puja Upadhyay, Ulhas Jadhav, Babaji Ghewade

**Affiliations:** 1 Department of Respiratory Medicine, Jawaharlal Nehru Medical College, Datta Meghe Institute of Medical Science (Deemed to be University), Wardha, IND

**Keywords:** transrectal-ultrasound guided prostate biopsy, prostate specific antigen, alkaline phosphatase, pulmonary tuberculosis, prostatic adenocarcinoma

## Abstract

Prostate carcinoma is one of the most common malignancies in the elderly male population in India as well as worldwide, and its incidence has been on the rise in the younger age groups as well. The annual incidence rate of prostate cancer in India ranges from 5.0 to 9.1 per 100,000 people. It commonly metastasizes to the bone, regional lymph nodes, and in rare cases, to the lung, liver, and brain. Pulmonary manifestations of metastatic prostate carcinoma are rare with pulmonary lesions being part of the initial pattern of metastasis in only 2% of prostate malignancies. We report the case of a 53-year-old male who presented with breathlessness and hemoptysis, which was initially diagnosed as pulmonary tuberculosis and later found to be a case of metastatic prostatic adenocarcinoma.

## Introduction

Prostate cancer is the most common non-cutaneous malignancy diagnosed in men, and its yearly incidence in India ranges from 5.0 to 9.1 per 1,00,000 people [1]. Tuberculosis is the most common differential diagnosis in pulmonary involvement of any form in India. Prostate cancer has a strong predilection for involving the skeletal system. After skeletal involvement, lung metastases and widespread lymphadenopathy are the most commonly seen outcomes. It is rare to encounter patients with symptomatic metastatic pulmonary lesions and lymphadenopathy due to prostate cancer at the initial presentation [2]. Although lung excavated metastases have been reported in the literature, initial diagnostic failure is frequent; 10% of metastatic lung adenocarcinomas with numerous cavitating nodules have been misdiagnosed as infectious diseases [3]. We report the case of a 53-year-old male with primary prostatic cancer presenting with pulmonary symptoms initially treated with anti-tubercular drugs.

## Case presentation

A 53-year-old male with no significant past medical history or comorbidities presented with breathlessness for two months, which had been insidious in onset and gradually progressed from Modified Medical Research Council (mMRC) grade II to mMRC grade III. The patient also had complaints of cough for one month, which was productive and accompanied by multiple episodes of blood-tinged expectoration. These symptoms had more recently been compounded by acute, midline lower back pain for 15 days. There was also associated anorexia and significant weight loss for the last three months prior to his presentation.

Upon admission, his physical examination revealed an overall cachexic appearance with a BMI of 16.3 kg/m^2^. His vitals parameters were as follows: pulse rate: 118/minute, blood pressure: 106/70 mmHg, respiratory rate: 24/minute, oxygen saturation on room air: 86% requiring 4-L/minute oxygen support. The patient also had pallor with no icterus, clubbing, or palpable lymphadenopathy. His respiratory examination revealed bilaterally diminished breath sounds and coarse crepitations on auscultation. The rest of his systemic examination was unremarkable.

Blood parameters revealed low hemoglobin, increased erythrocyte sedimentation rate (ESR), and elevated alkaline phosphatase (ALP). The rest of the laboratory parameters including kidney function tests and liver function tests were within normal limits (Table 1).

**Table 1:** Laboratory investigations of the patient

Investigations	Values	Biological reference range
Hemoglobin	8.8 g/dl	13-15 g/dl
Mean corpuscular volume	73.7 fL	79-100 fL
Total leukocyte count	8,700/cumm	4,000-11,000/cumm
Platelet count	3,23,000/cumm	1,50,000-4,50,000/cumm
Erythrocyte sedimentation rate	63 mm/hr	0-22 mm/hr
Serum sodium	136 mmol/L	135-145 mmol/L
Serum potassium	3.8 mmol/L	3.5-4.5 mmol/L
Serum creatinine	0.4 mg/dl	0.3-1.2 mg/dl
Alkaline phosphatase	1026 U/L	38-126 U/L
Total bilirubin	0.8 mg/dl	0.2-1.3 mg/dl

**Figure 1:**
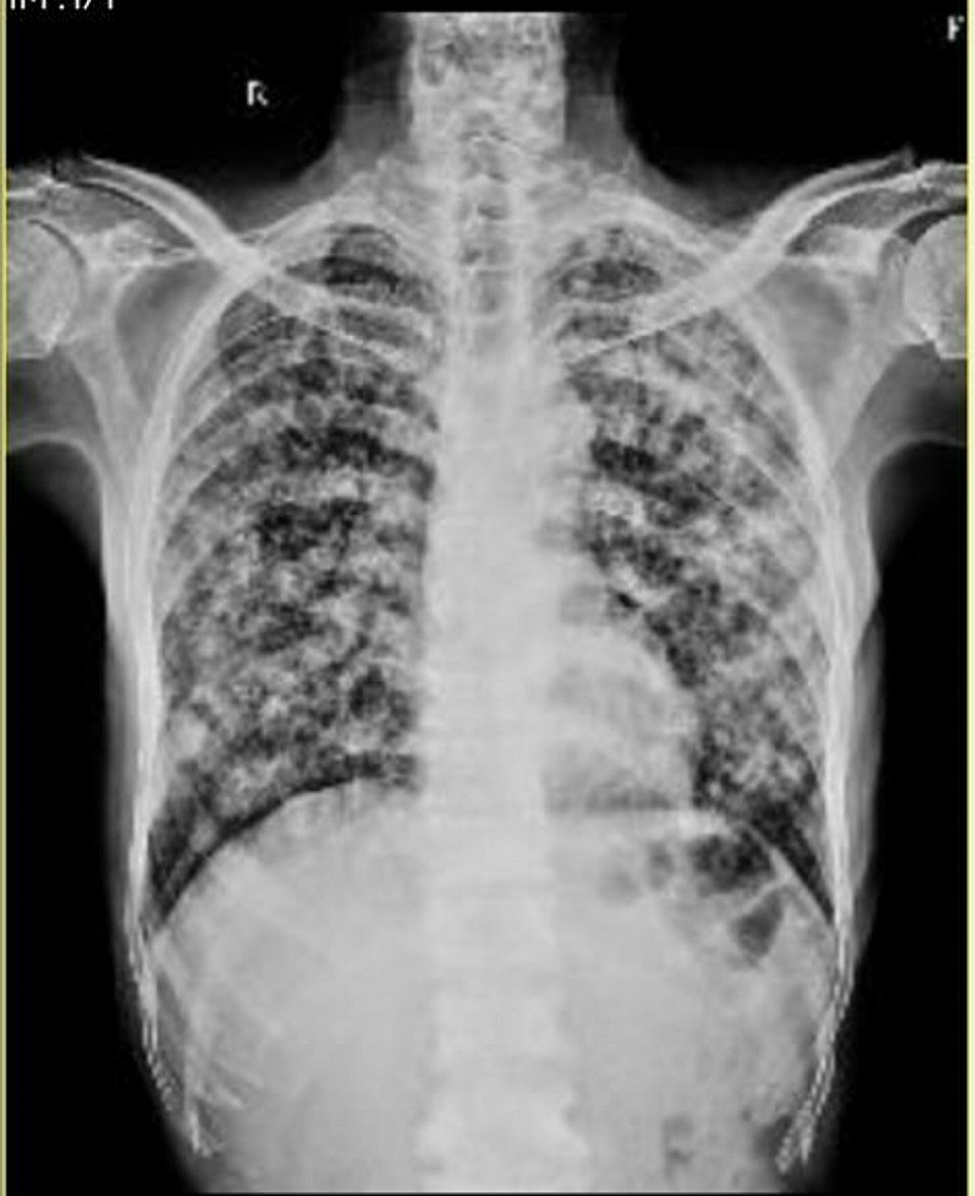
Chest X-ray posteroanterior view s/o extensive bilateral ill-defined fluffy opacities throughout the lung fields

The patient's chest X-ray showed extensive bilateral ill-defined fluffy opacities throughout the lung fields (Figure 1).

The patient’s further workup was based on a presumptive possibility of pulmonary tuberculosis, which included sputum acid-fast bacilli (AFB) and GeneXpert and tuberculin skin tests (Table 2).

**Table 2:** Workup for pulmonary tuberculosis

Workup	Result
Sputum acid-fast bacilli (AFB)	Negative
GeneXpert	Negative
Tuberculin skin test	Negative

Based on the above results, the diagnosis of smear-negative pulmonary tuberculosis was made and the patient was empirically started on anti-tubercular drugs in a fixed-dose combination of isoniazid, rifampicin, pyrazinamide, and ethambutol. 

**Figure 2:**
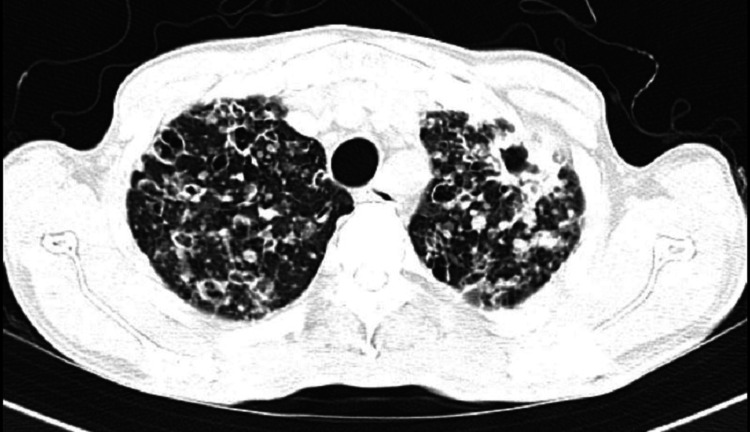
CECT of the thorax The image revealed multiple nodular opacities of varying sizes showing mild post-contrast enhancement with peri-nodular consolidations along with central cavitation located diffusely in bilateral lung fields CECT: contrast-enhanced computed tomography

The patient did not respond to anti-tubercular treatment after a period of two weeks and continued to have worsening symptoms and a rising trend of alkaline phosphatase (ALP) over the course of the hospital stay. In light of the persistence of bony pain in the form of back pain since the initial presentation and raised ALP along with pulmonary manifestations, he was subsequently worked up for multiple myeloma. However, urine B and J protein was negative with no other supportive diagnostic features. A contrast-enhanced CT (CECT) of the thorax was done, which demonstrated multiple nodular opacities of varying sizes showing mild post-contrast enhancement with peri-nodular consolidations along with central cavitation located diffusely in the bilateral lung fields as well as multiple bony sclerotic lesions (Figure 2).

For obtaining a definitive diagnosis, bronchoscopy was performed and broncho-alveolar lavage (BAL) was collected and sent for AFB and GeneXpert, which returned negative with inconclusive cytology.

**Figure 3:**
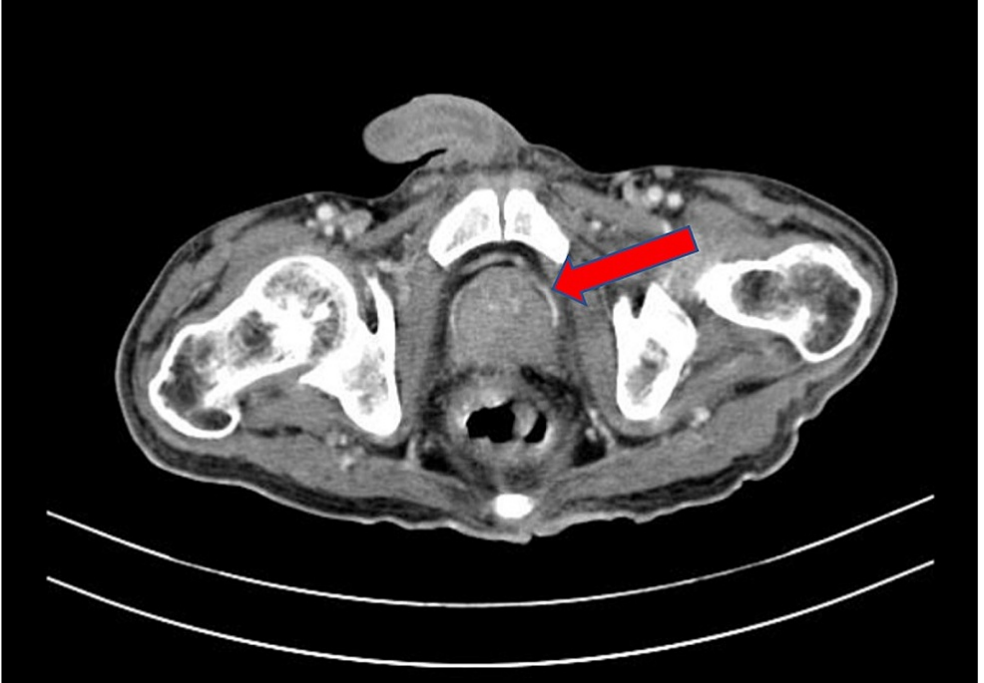
CECT abdomen and pelvis showing enlarged prostate (arrow) CECT: contrast-enhanced computed tomography

**Figure 4:**
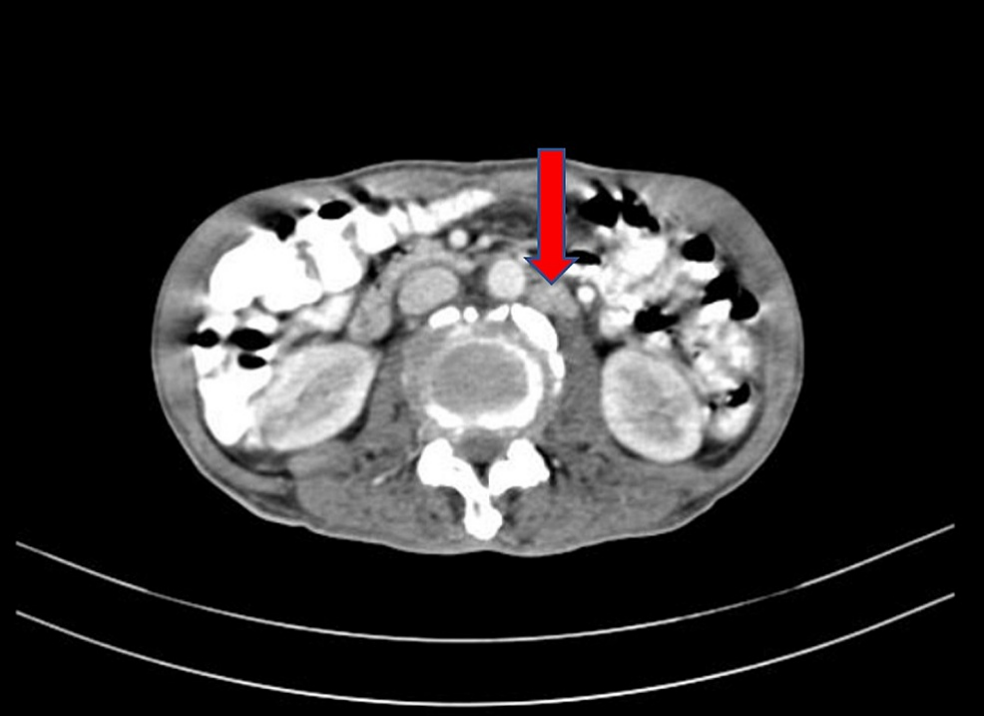
CECT abdomen and pelvis showing retroperitoneal lymph nodes (arrow) CECT: contrast-enhanced computed tomography

**Figure 5:**
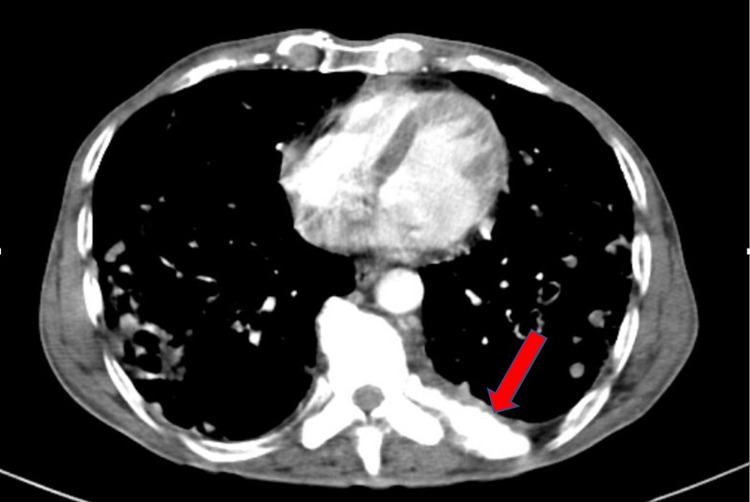
CECT abdomen and pelvis showing vertebral sclerotic lesion (arrow) CECT: contrast-enhanced computed tomography

Keeping a possibility of metastatic involvement of the lungs in mind and to search for the primary, a CECT of the abdomen was performed, which showed retroperitoneal lymphadenopathy with an enlarged prostate and multiple sclerotic bony lesions highly suspicious of malignancy (Figures 3, 4, 5).

**Figure 6:**
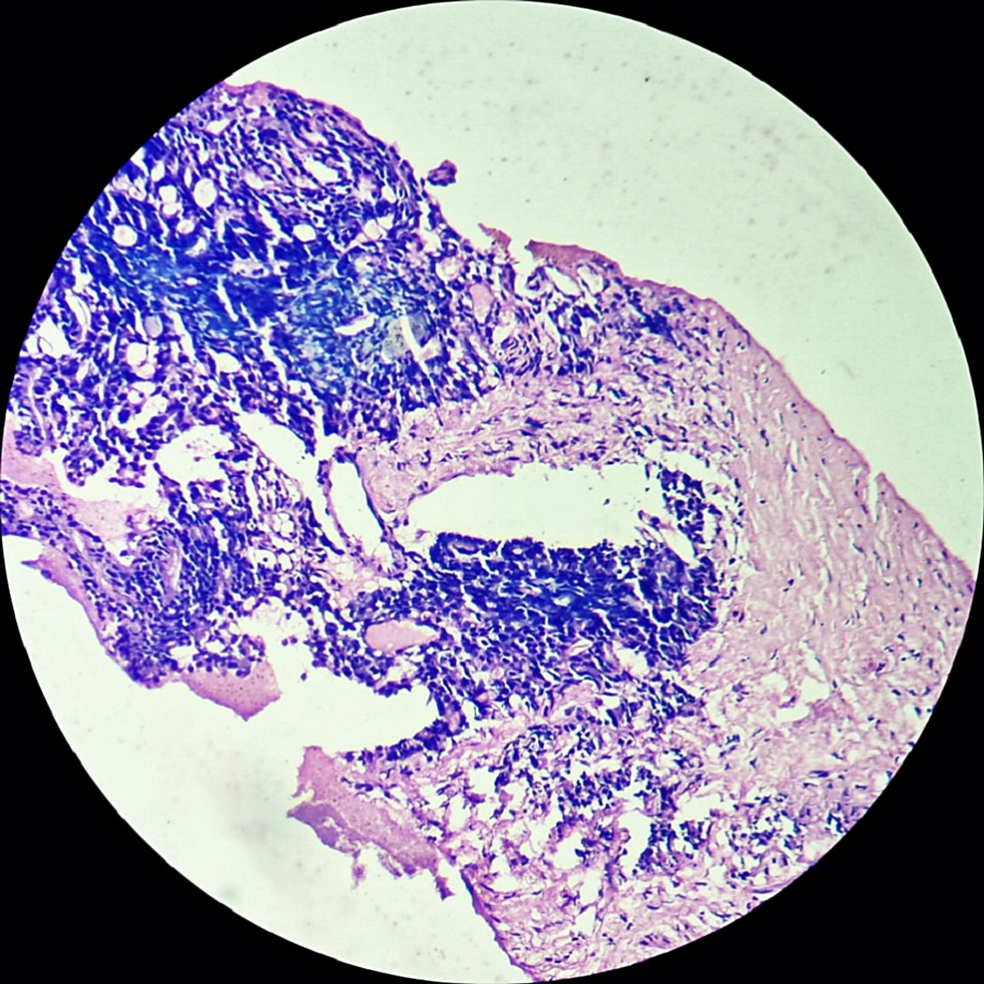
H&E section showing infiltrates of adenocarcinoma of the prostate (4X) H&E: hematoxylin and eosin

With prostatic carcinoma now high on the differential, a serum prostate-specific antigen (PSA) was done, which was found to be significantly elevated at 1446 ng/ml (normal value: below 4.0 ng/ml). For confirmatory testing, a transrectal-ultrasound-guided prostate biopsy was performed, and it revealed pathology consistent with an adenocarcinoma of the prostate (Figure 6).

## Discussion

Prostate carcinoma is one of the most common malignancies in older men and one of the leading causes of mortality related to malignancy globally. The lungs are the second or third most frequent metastatic site after bone and/or lymph node secondary to carcinoma prostate [4,5]. At the time of initial diagnosis, 5-27% of patients have clinically evident lung metastases. In 2% of prostate cancers, pulmonary lesions were found to be part of the initial pattern of metastasis [6]. The most common radiological patterns of pulmonary metastases in advanced prostate cancer are lymphangitic or nodular, with the former being more common since it appears to be the result of direct invasion of lung lymphatics, whereas the nodular pattern is the result of hematogenous dissemination [4,6].

In this report, we documented a noteworthy case of adenocarcinoma prostate where lung and bony metastases and lymphadenopathy were seen during the initial presentation without any concomitant lower urinary tract symptoms (LUTS) or any multiorgan involvement. Another distinctive attribute of our report pertains to the aberrant pulmonary metastatic pattern seen in the form of cavitation in metastatic nodules.

In contrast to the 9% frequency of cavitation seen originally in bronchogenic carcinoma, the frequency of cavitation in metastatic nodules observed on a radiograph is roughly 4% [7]. Cavitary form of metastasis is frequently seen in metastatic squamous cell carcinoma as compared to adenocarcinoma [8]. The precise basis of cavitation is undetermined, but it is thought to be either tumor necrosis or a check valve mechanism that arises as a result of tumor infiltration into the bronchial system [7,9]. Excavated lesions in the lung parenchyma are an atypical radiological presentation of pulmonary metastasis, and the most common differential diagnostic hypotheses for these appearances include bacterial or fungal infections, rheumatoid nodules, vasculitis processes, lymphomas, and, most pertinently, presumptive suspicion of extensive tuberculosis in a country like India where tuberculosis is considered a grave public health concern [3,10].

In this vein, our findings clearly suggest that conditions like tuberculosis and various forms of cancers might mimic each other and have aberrant clinical and radiological manifestations. Misdiagnosis can be deleterious in both ways; in the case of underlying or co-existing tuberculosis, initiating immunosuppressive therapy can lead to infectious dissemination and, in some cases, death; or, as in our case, empirical initiation of anti-tubercular treatment may deviate us from unraveling the underlying existing diagnosis. Our vigilance in terms of observing the unresponsiveness to anti-tubercular treatment and persistent efforts to reach the diagnosis had stumped us with the indisputable conclusion. 

On initial diagnosis, the above-mentioned clinical signs are relatively uncommon, and the absence of urinary symptoms may obscure the possibility of a primary adenocarcinoma prostate presenting as metastatic lung disease.

## Conclusions

This case report emphasizes that clinicians and radiologists should be mindful of the possibility of underlying carcinoma prostate in patients presenting with pulmonary manifestations and concomitant atypical radiological metastatic patterns. When encountering such cases, they are best approached with the aid of a multidisciplinary team. Lastly, we need to be watchful regarding the myriad manifestations of metastatic prostate carcinoma and resist the temptation of premature diagnostic closure.
